# Isolation, Characterization and Neuroprotective Activity of Folecitin: An In Vivo Study

**DOI:** 10.3390/life11080825

**Published:** 2021-08-12

**Authors:** Umar Farooq, Taous Khan, Shahid Ali Shah, Md. Sanower Hossain, Yousaf Ali, Rahim Ullah, Naila Raziq, Muhammad Shahid, Raffaele Capasso

**Affiliations:** 1 Department of Pharmacy, Abbottabad Campus, COMSATS University Islamabad, Abbottabad 22060, Pakistan; FA14-R60-006@cuiatd.edu.pk; 2 Department of Chemistry, Sarhad University of Science and Information Technology, Peshawar 25000, Pakistan; shahid.chemistry@suit.edu.pk (S.A.S.); yousaf.chemistry@suit.edu.pk (Y.A.); 3 Neuromolecular Medicine Research Center, Ring Road, Peshawar 25000, Pakistan; 4 Department of Biomedical Science, Kulliyyah of Allied Health Sciences, International Islamic University Malaysia, Kuantan 25200, Malaysia; 5 Faculty of Science, Sristy College of Tangail, Tangail 1900, Bangladesh; 6 Department of Pharmacy, University of Peshawar, Peshawar 25120, Pakistan; rphrahimullahstd@uop.edu.pk; 7 Department of Pharmacy, Sarhad University of Science and Information Technology, Peshawar 25000, Pakistan; naila.fls@suit.edu.pk (N.R.); shahid.fls@suit.edu.pk (M.S.); 8 Department of Agricultural Sciences, University of Naples Federico II, 80055 Portici, Italy

**Keywords:** alcohol, flavonoid, medicinal plant, neuroinflammation, neuroprotection

## Abstract

Neurodegenerative diseases (NDs) extend the global health burden. Consumption of alcohol as well as maternal exposure to ethanol can damage several neuronal functions and cause cognition and behavioral abnormalities. Ethanol induces oxidative stress that is linked to the development of NDs. Treatment options for NDs are yet scarce, and natural product-based treatments could facilitate ND management since plants possess plenty of bioactive metabolites, including flavonoids, which typically demonstrate antioxidant and anti-inflammatory properties. *Hypericum oblongifolium* is an important traditional medicinal plant used for hepatitis, gastric ulcer, external wounds, and other gastrointestinal disorders. However, it also possesses multiple bioactive compounds and antioxidant properties, but the evaluation of isolated pure compounds for neuroprotective efficacy has not been done yet. Therefore, in the current study, we aim to isolate and characterize the bioactive flavonoid folecitin and evaluate its neuroprotective activity against ethanol-induced oxidative-stress-mediated neurodegeneration in the hippocampus of postnatal day 7 (PND-7) rat pups. A single dose of ethanol (5 g/kg body weight) was intraperitoneally administered after the birth of rat pups on PND-7. This caused oxidative stress accompanied by the activation of phosphorylated-c-Jun N-terminal kinase (p-JNK), nod-like receptor family pyrin domain containing 3 (NLRP3), apoptosis-associated speck-like protein (ASC), and cysteine-aspartic acid protease-1 (caspase-1) proteins to form a complex called the NLRP3-inflammasome, which converts pro-interleukin 1 beta (IL-1B) to activate IL-1B and induce widespread neuroinflammation and neurodegeneration. In contrast, co-administration of folecitin (30 mg/kg body weight) reduced ethanol-induced oxidative stress, inhibited p-JNK, and deactivated the NLRP3-inflammasome complex. Furthermore, folecitin administration reduced neuroinflammatory and neurodegenerative protein markers, including decreased caspase-3, BCL-2-associated X protein (BAX), B cell CLL/lymphoma 2 (BCL-2), and poly (ADP-ribose) polymerase-1 (PARP-1) expression in the immature rat brain. These findings conclude that folecitin is a flavone compound, and it might be a novel, natural and safe agent to curb oxidative stress and its downstream harmful effects, including inflammasome activation, neuroinflammation, and neurodegeneration. Further evaluation in a dose-dependent manner would be worth it in order to find a suitable dose regimen for NDs.

## 1. Introduction

One of the common and growing global health burdens, particularly in the elderly, is neurodegenerative diseases (NDs) that inexorably progress to severe disability and death. NDs are linked with other risk factors such as the presence of the *ApoE* e4 allele, cerebrovascular diseases, hyperlipidemia, smoking, diabetes, obesity, and traumatic brain injury [1]. These risk factors impose additional health and economic burden. Chronic consumption of alcohol induces the development of NDs as it changes many biochemical and physiological actions in the central nervous system (CNS), of which some alterations pertain to specific neurotransmitter system changes and intricate signaling pathways [2]. Moreover, alcoholic beverage consumption during pregnancy can produce a wide range of toxic effects, including teratogenicity and lethality to the prenatal fetus, leading to neuronal damage, abnormal childbirth, and postnatal mental health problems [3,4].

Among many established mechanisms of alcohol-induced NDs, oxidative stress has received much more attention in the last few years due to excessive ethanol ingestion producing an increasing amount of reactive oxygen species (ROS) and, to a lesser extent, reactive nitrogen species (RNS); it suppresses antioxidant defense mechanisms, which inactivate the ROS system, thereby resulting in oxidative stress (OXS) and/or nitrosative stress (NSS) [3,5,6]. Because of OXS, high ethanol consumption involves the depletion of beneficial glutathione (GSH) levels and the elevation of harmful malondialdehyde, hydroxyl-ethyl radical, and hydroxynonenal protein adducts, resulting in serious cell and tissue malfunction and the progression of neuroinflammation, which can be the cause of NDs [7]. Moreover, ethanol smoothly overcomes the blood–brain barrier and induces OXS that accelerates neuronal damage by stimulating BAX and endorsing the cleavage of caspase-3 [8,9]. Since fetal brain development requires a low oxygen environment and produces limited antioxidants [5], their CNS is vulnerable to maternally consumed alcohol-mediated ROS-induced OXS [10].

Ethanol is one of the most abused drugs. As maternal exposure to ethanol impairs many neuronal functions, which lead to cognition and behavioral abnormalities, broadly labeled as fetal alcohol syndrome (FAS) [11], animal models have been used for NDs induced by exposure to ethanol to understand the underlying mechanisms and to investigate potential therapeutics [12,13,14]. Even though, arguably, no animal model of NDs fully phenocopies human disease, many models recapitulate the initial proteinopathy or other pathological features linked to the human disorder [15]. Among many animal models, rodents are predominantly used to induce a specific characteristic of ND, as per the experimental objective. Rodents exposed to a few hours of a single exposure to ethanol at the developmental stage have significant neuronal loss throughout the forebrain [16], which might continue to the mature stage [17]. The hippocampus in the developing brain is important for learning and memory and spatial navigation, emotional behavior, and the regulation of hypothalamic functions. However, it is one of the most sensitive organs to ethanol exposure, which causes hippocampal tissue damages [18]. Thereby, hippocampal tissue abnormalities disrupt cell density, inhibit presynaptic glutamate release and binding affinity, and, ultimately, cause memory, learning, and behavioral impairments [19].

The precise mechanisms leading to neuronal loss in the development of the brain while exposed to ethanol are not yet fully understood. Ethanol induces NDs by producing ROS and through cascades of devastating effects on the neuronal cells. In short, ethanol can block the *N*-methyl-D-aspartic acid or *N*-methyl-D-aspartate (NMDA) receptor, inhibit phosphatidylinositol-4,5-bisphosphate 3-kinase (PI3K), and inhibit downstream signaling molecules, including the suppression of v-akt murine thymoma viral oncogene (AKT) and the activation of serine/threonine protein kinase-3 (GSK3) [3,19,20,21].

Although the treatment opportunity to cure ND conditions is not available, there is an increasing range of available therapeutic and supportive options. As therapeutic options, natural products such as phytocompounds have gained esteemed popularity in recent years [22,23,24]. Currently, about 75–80% of people of developing countries and about 25% of people of developed countries rely either directly or indirectly on medicinal plants for the first line of treatment [25,26,27] as they are considered safer than synthetic drugs [28,29]. Since natural products are better models, with ideal pharmacokinetics/pharmacodynamics properties, and about 80% of drugs are either natural products or analogs mimicking them, with a steadily increasing approval rate by FDA, they are investigated widely for treatment opportunities for the development of polypharmacological drugs for multifactorial disorders, including infectious diseases, cancers, and NDs [30]. Moreover, the indication of the importance and therapeutic efficacy of medicinal plants or natural products in religious scripts make them more attractive to researchers when establishing the validity of traditional use to the scientific community [31,32]. Antioxidants extracted from medicinal plants and fruits are ideal candidates to protect the neuronal damage caused by OXS [23].

*Hypericum oblongifolium* from the *Hypericaceae* family is native to Eurasia. It grows at elevations of 4000–6000 m in the Himalayas, China, and northern parts of Pakistan, including Kashmir, Hazara, and Murree Hills [33,34]. It is a flowering plant (6–12 m height), traditionally used for hepatitis, gastric ulcers, external wounds, and other gastrointestinal disorders [35]. *H. oblongifolium* has been studied for its significant antidepressant and antinociceptive activities [33,34]. It also possesses potent in vitro anti-inflammatory, anti-glycation, antioxidant, and anti-lipid peroxidation activities [36]. Additionally, it shows anti-proliferative solid activity on HT-29 human colon adenocarcinoma [37]. Although this plant has potential medicinal uses and pharmacological properties, the investigation of the phytochemicals and their pharmacological activities is still limited. In 2015, Raziq et al. [33] reported several novel flavonoids from *H. oblongifolium* and demonstrated potent antioxidant properties. Flavonoids can cross the blood–brain barrier and affect different mechanisms involved in the progressions of neuroinflammation and neurodegeneration in the CNS [38]. Even though *H. oblongifolium* possesses potent anti-inflammatory and antioxidant activities, to our best knowledge, no study has yet been conducted to evaluate the effects of isolated pure flavonoid on OXS-induced NDs in a rat model. Therefore, this study is designed to isolate a pure flavonoid and to evaluate its possible neuroprotective effect against ethanol-induced ROS, inflammation, and neuronal apoptosis on the brain of postnatal day 7 (PND-7) rat pups.

## 2. Materials and Methods

### 2.1. Chemicals

Ethanol, methanol, ethyl acetate, n-hexane, polyvinylidene fluoride (PVDF) membrane, phosphate-buffered saline tablets, RNAwait solution, tissue protein extraction (T-PER) kit, protein assay dye, sample buffer (2X Laemmli), trizma base, acrylamide, bis-acrylamide, sodium dodecyl sulfate (SDS), ammonium persulfate (APS), tetramethylethylenediamine (TEMED), glycine, skim milk, KCl, NaCl, Tween 20 and H_2_O_2_, guaiacol, phenazine methosulphate, glacial acetic acid, sulfosalicylic acid, DTNB ascorbic acid, trichloroacetic acid, and thiobarbituric acid were purchased from Sigma-Aldrich Chemical Co. (St. Louis, MO, USA). All the chemicals and reagents were stored at the required temperature according to the material safety and data sheet for experimental purposes.

### 2.2. Bioactive Compound Isolation

#### 2.2.1. Plant Materials

*H. oblongifolium* was collected from Thandiyani, Abbottabad, Khyber Pakhtunkhwa, Pakistan, and authenticated (voucher specimen no. Atk/102/2018) at the Department of Botany, Government Post Graduate College, Attock City, Pakistan.

#### 2.2.2. Folecitin Isolation and Characterization

Folecitin was isolated from fresh leaves of *H. oblongifolium* and characterized according to Raziq et al. [33]. Briefly, fresh leaves (15 kg) were pulverized and macerated in 70% methanol for 14 days, with constant stirring using a steel rod. After this extraction period, the solvent was settled, poured, and filtered with Whatman 42 filter paper with a 2.5 µm pore size (catalog # Whatman 1442-042). The crude solvent was concentrated under vacuum at 45 °C using a rotary evaporator (Büchi Rotavapor R-210, New Castle, DE, USA). To obtain the maximum concentration of methanolic crude extract, the same procedure was repeated for an additional seven days using fresh 70% methanol. This crude extract was mixed with 1 L of distilled water in the fractionating column and fractionated using ethyl acetate.

About 400 g of ethyl acetate fraction was exposed to column chromatography (CC) over silica gel mesh size 230 (Merck, Germany) to isolate folecitin. The column length and diameter was 1000 and 40 mm respectively. The column was run under gravitational force, and the fraction was eluted with an n-hexane:ethyl acetate (2:8) solvent system ratio and a flow rate of 2 mL/min. Finally, a total of 150 fractions (10 mL each) were obtained based on the fingerprinting analysis of thin-layer chromatography (TLC; silica gel 60 PF254, Merck, Germany). After the pooling of similar fractions, 16 major fractions were obtained. Fraction 14 (1.2 g) was subjected to the CC (400 × 10 mm) over flash silica gel (ethyl acetate:chloroform, 1:1) under gravitational force. The reanalysis of this fraction in TCL resulted in 12 major fractions (10 mL each). Fractions 5–9 were combined and led to the isolation of a compound (70 mg).

Folecitin was visualized by spraying solid iodine and cerium sulfate (CeSO4), followed by a heating process. The structure of folecitin was confirmed with ^1^H and ^13^C NMR, HMBC, COSY, and HSQC spectra with Bruker spectrometers (Billerica, MA, USA) (Avance Av 500, 600/150 MHz).

### 2.3. Neuroprotective Efficacy of Folecitin

#### 2.3.1. Animals Used in the Experiment

Sprague–Dawley PND-7 rat pups (~18 g each) were obtained from the Veterinary Research Institute, Peshawar, KPK, Pakistan. They were moved to the experimental lab with extensive care without allowing any external effects on the pups. They were kept in rat cages group-wise, with their mother in the animal lab at a controlled room temperature (25 ± 2 °C) and humidity (60–65%). The pups’ mothers were allowed to access food and water ad libitum. For animal care and treatment, we followed the guidelines of the UK Animals (Scientific Procedures) Act 1986 [39]. The experimental procedures on animals were approved (Ref. No. NMMRC/03/2019) on 11 September 2019 by the ethics committee of the Neuro Molecular Medicine Research Center (NMMRC), Ring Road, Peshawar, Pakistan.

#### 2.3.2. Experimental Design and Approach

**Figure 1:**
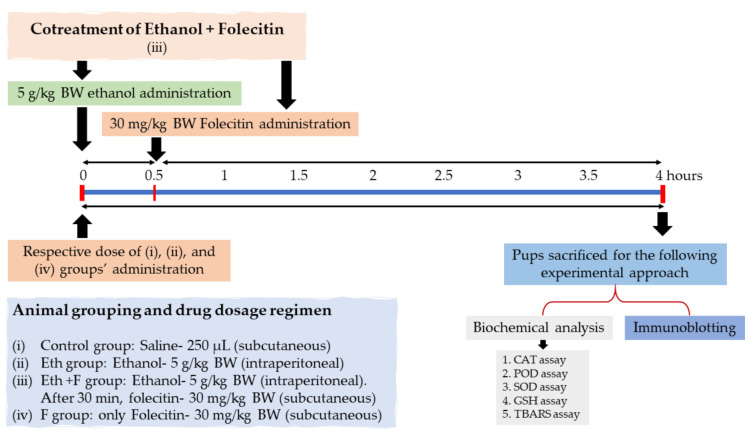
Experimental design, animal grouping (*n* = 6 each group), dosage regimen for the drug, and the biochemical experimental approach for the whole study. BW: body weight, CAT: catalase, POD: peroxidase, SOD: superoxide dismutase, GSH: glutathione, TBARS: thiobarbituric acid reactive substances.

The following experimental design and approach (Figure 1) were set to accomplish the hypothesis of this study. Only male PND-7 rat pups were chosen in this study to evade ambiguous sex-dependent differences. PND-7 rat pups were randomly divided into four groups (*n* = 6): PND-7 rat pups were (i) treated with a single dose of saline (250 µL) as a vehicle (control group); (ii) treated with a single dose of ethanol (5 g/kg body weight), injected intraperitoneally (i.p.) (Eth group); (iii) treated with a single dose of folecitin, injected subcutaneously at a dose of 30 mg/kg body weight after 30 min of i.p. administration of ethanol at a dose of 5 g/kg body weight (Eth + F group); and (iv) treated with a single dose of the only folecitin, injected subcutaneously at a dose of 30 mg/kg body weight (F group). After this, all experimental groups were kept under observation for a total of 4 h.

#### 2.3.3. Protein Extraction for Biochemical Analysis and Immunoblotting

At the end of the experiment, the PND-7 rat pups were sacrificed, and the brains were collected immediately; the hippocampus was separated carefully, and the tissue was frozen on dry ice and store at −80 °C. The brain samples were homogenized using a tissue protein extraction reagent (T-PER) with a phosphatase inhibitor and protease inhibitor cocktail and then centrifuged at 10,000× *g* at 4 °C for 5 min. The supernatants were collected and stored at −80 °C for further use.

#### 2.3.4. Biochemical Analysis

##### Catalase Assay (CAT)

For the CAT, 3 mL of the mixture contained 2.5 mL of phosphate buffer saline (PBS; 50 mM) at pH 5.0, 100 µL of H_2_O_2_ (5.9 mM), and 100 μL of brain supernatant. The change in absorbance of the reaction blend was measured at an interval of one minute at 240 nm. The alteration in absorbance of 0.01 units/min was measured as one unit of activity.

##### Peroxidase Assay (POD)

The reaction blend for the peroxidase assay consisted of 300 µL of H_2_O_2_ (40 mM), 2500 µL of PBS (50 mM) at pH 5.0, 100 µL of guaiacol (20 mM), and 1000 µL of brain homogenate supernatant. The alteration in absorbance of the reaction merger was noted at a one-minute interval at 470 nm. One unit of POD action was regarded as the alteration in absorbance of 0.01 units/min.

##### Superoxide Dismutase Assay (SOD)

To estimate the SOD action, the reaction blend contained 1200 µL of sodium pyrophosphate buffer (0.052 mM, pH 7.0), 100 µL phenazine methosulphate (186 µM), and 300 µL of supernatant brain homogenate. To start the enzymatic response, 200 µL of reduced nicotinamide adenine dinucleotide (NADH) (780 µM) was incorporated into the reaction mixture, and, after 1 min, 1000 µL of glacial acetic acid was added as the discontinuing agent. The amount of chromogen formed was determined by taking the absorbance of the reaction mixture (at 560 nm); outcomes were noted as units per mg of protein.

##### Reduced Glutathione Assay (GSH)

To estimate reduced GSH levels, 1 mL of the brain (homogenate) was used to precipitate proteins by adding an equivalent volume of 4% sulfosalicylic acid solution. The reaction blend was incubated at 4 °C for 1 h, later centrifuged for 20 min at 4 °C at 1200× *g*. The reaction blend contained 2.7 mL of PBS (0.1 M) at pH 7.4, 100µL of centrifuged aliquot, and 200 µL of 100 mM Ellman’s reagent (5,5′-dithiobis-(2-nitrobenzoic acid) (DTNB). The absorbance of the reaction blend was taken immediately at 412 nm. The effects of reduced GSH were communicated as µM/g tissue.

##### Approximation of Lipid Peroxidation

The thiobarbituric acid reactive substance (TBARS) assay was used to determine lipid peroxidation. The reaction mixture was 1 mL, which contained 580 µL of PBS (0.1 M; pH 7.4), 200 µL of ascorbic acid (100 mM), 200 µL of supernatant brain homogenate, and 20 µL of ferric chloride (100 mM). The reaction blend was incubated for one hour in pulsating water bath maintained at 37 °C. To stop the reaction, 1 mL of trichloroacetic acid (10%) solution was added. Later, 1 mL of thiobarbituric acid (0.67%) was added to the tubes, and the tubes were positioned in a hot water bath (95 °C) for 20 min, then rapidly moved to the crushed ice and centrifuged for 10 min at 2500 rpm. The quantity of lipid peroxidation made in every section was determined by calculating the absorbance of the supernatant on a UV spectrophotometer at 535 nm. The outcomes were stated as nM TBARS/min/mg of tissue at 37 °C (the TBARS molar extinction coefficient is 1.56 × 10^5^ M^−1^cm^−1^).

### 2.4. Western Blotting

Western Blot analysis was performed according to the previously described methods, with minor modifications [22,23,24]. Briefly, the extracted proteins from the hippocampus of PND-7 were analyzed quantitatively using BioRad protein assay solution. The quantity of protein was assessed and analyzed through SDS-PAGE. An equal amount of protein (30 µg per sample) was loaded on a 10–15% SDS-PAGE gradient gel under reduced conditions. A 10–245 kDa range pre-stained protein marker (GangNam-STAIN™, iNtRon Biotechnology, Inc., Seongnam, Korea) was used throughout the study to determine the desired protein’s molecular weight.

A wide range of mouse-derived antibodies (Santa Cruz Biotechnology, Santa Cruz, CA, USA) was used (1:500) to detect different proteins. including anti-poly (ADP-ribose) polymerase 1 (PARP-1) anti-tumor necrosis factor α (TNFα), anti-nuclear factor kappa-light-chain-enhancer of activated B-cells (NF-kB), anti-Jun N-terminal kinase p-JNK, anti-NLRP-3, anti-caspase-1, anti-interleukin-1-beta (IL-1β), anti-apoptosis-associated speck-like protein (ASC), anti-BCL-2-associated X protein (BAX), and anti-B cell CLL/lymphoma 2 (BCL-2). To lessen the binding of non-specific proteins, 5% (*w/v*) skim milk was used to block the membranes [22,40]. Incubation with the primary antibody was performed for 24 h at 4 °C. After rinsing the blots, horseradish-peroxidase-conjugated goat anti-mouse secondary antibodies (IgG-HRPs) (Santa Cruz Biotech 1:1000) were incubated with the blots for two hours at room temperature. According to the manufacturer’s instructions, an ECL (Amersham Pharmacia Biotech, Uppsala, Sweden) detection reagent was used for visualization after using membrane-derived secondary antibodies. The X-ray films were scanned, and the optical densities were analyzed by densitometry using the computer-based Sigma Gel program version 1.0 (SPSS, Chicago, IL, USA). The list of primary and secondary antibodies, along with catalog numbers, is shown in Table 1.

### 2.5. Statistical Analysis

Results are presented as mean ± standard deviation (SD). Multiple group means of parametric data sets were compared using one-way analysis of variance (ANOVA) after it was determined that the data conformed to a normal distribution with equal variances. If an overall significance was found, Tukey’s multiple-comparison post hoc test was applied using GraphPad Prism 5 (GraphPad Software Inc. San Diego CA, USA). A *p* < 0.05 value was considered statistically significant.

## 3. Results

### 3.1. Folecitin’s Yield

After being re-chromatographed and eluted with n-hexane:ethyl acetate (2:8), 400 g of ethyl acetate fraction finally yielded 70 mg of yellowish-brown crystalline solid powders, equivalent to the yield of 175 mg/kg crude extract of fresh leaves (Table 2).

### 3.2. Folecitin’s Characterization

**Figure 2:**
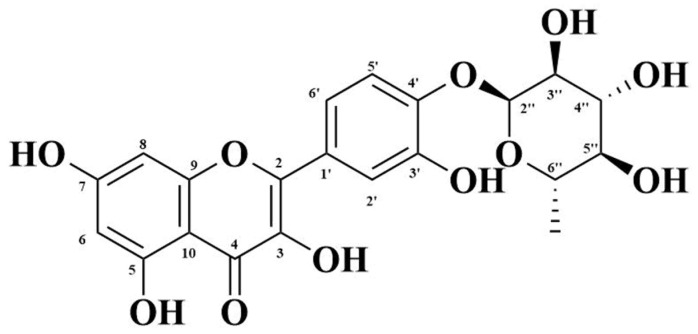
Structure of folecitin.

The characterization of this crystal powder was done by ^1^H and ^13^C NMR, followed by HMBC, COSY, and HSQC spectra for the confirmation of the compound’s structure (Table 2). The compound was determined as folecitin, IUPAC: 3,5,7-trihydroxy-2-[3-hydroxy-4 (3,4,5-trihydroxy-6-methyltetrahydro-2H–pyran–2-yloxy) phenyl]-4H-chromen-4-one (Figure 2). The mass and spectral data is supplied in supplementary data Figures S1–S10, while the physical characteristics of folecitin are given below in Table 2.

#### 3.2.1. Electrospray Ionization Mass Spectrometry (ESI–MS) Spectrum

[M+H]1+ peak showed 449.11 amu (Figure S1). The m/z (mass-to-charge ratio) was 448.10 (100.0%), 449.10 (22.7%), 450.11 (2.5%), and 450.10 (2.3%). The m/z ratio corresponded to the molecular formula for C_21_H_20_O_11._

#### 3.2.2. Infrared Spectroscopy (IR) Spectrum

The IR spectrum exhibited absorption bands (cm^−1^, KBr): v (O-H) is 3245, v (C=O) is 1655, and v (C=C) is 1608 (Figure S2).

#### 3.2.3. Nuclear Magnetic Resonance Spectroscopy (NRM) Spectrum

^1^H NMR (δ in ppm, J in Hz, DMSO-d6, 500 MHz): Aromatic protons: C-6 (δ = 6.19, 1H, d, J = 2.0 Hz), C-8 (δ = 6.38, 1H, d, J = 2.0 Hz), C-2′ (δ = 7.30, 1H, d, J = 2.0 Hz), C-5′ (δ = 6.90, 1H, d, J = 8.0 Hz), C-6′ (δ = 7.25, 1H, dd, J = 8.0, 2.0 Hz); carbonylic protons: C-3″ (δ = 4.59, 1H, dd, J = 3.5, 1.5 Hz), H-4″ (δ = 3.23, 1H, m), H-5″ (δ = 3.50, 1H, dd, J = 9.5, 3.5 Hz); methyl group: C-7″ (δ = 0.807, 3H, d, J = 6.0 Hz) (Figures S2 and S3).

^13^C NMR (δ in ppm, DMSO-d6, 150 MHz): Quaternary -OH groups: C-3 (134.6), C-5 (161.7), C-7 (145.6), C-3′ (145.6), C-3″ (70.44), C-4″ (71.56), C-5″ (70.73); C=O group: C-4 (178.14); methyl group: C-6″ (17.9); secondary carbon group: C-2 (156.9), C-6 (99.1), C-8 (94.02), C-2′ (116.04), C-4′ (148.83), C-5′ (115.85), C-6′ (121.11), C-2″ (102.22); tertiary carbon: C-10 (104.5), C-1′ (121.5) (Figure S5).

**Figure 3:**
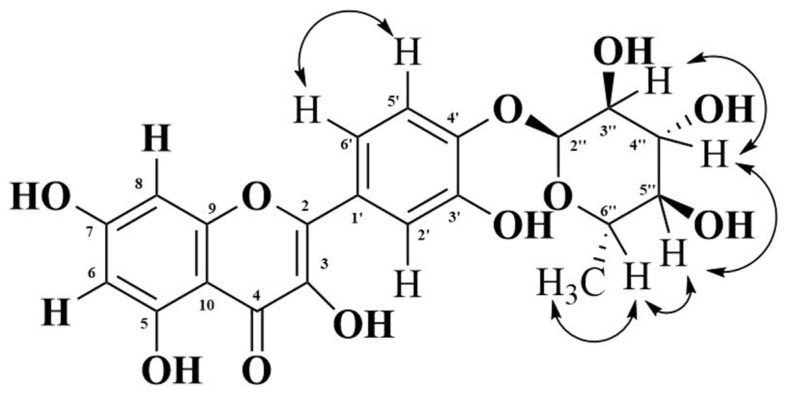
Correlation between hydrogen atoms at the adjacent carbons. Both end arrows show the COSY cross peak.

The ^1^H-^1^H correlation spectroscopy (COSY) spectrum shows correlations between protons that are coupled at the adjacent carbons (Figure S8). Protons at C-5′ and C-6′ showed a COSY cross peak. Protons between C-3″ and C-4″, C-4″ and C-5″, C-5″ and C-6″, and C-6″ and C-7″ C-H protons at δ 4.59 (H-3″) showed COSY cross-peaks; the proton at δ 3.23 (H-4″) showed COSY cross-peaks, which, in turn, showed COSY cross-peaks with H-5″ (δ 3.50). A relay of the COSY cross-peaks between methine (C-6″) and methyl (C-7″) protons suggests that all of them are in the same cycle and ring (Figure 3).

**Figure 4:**
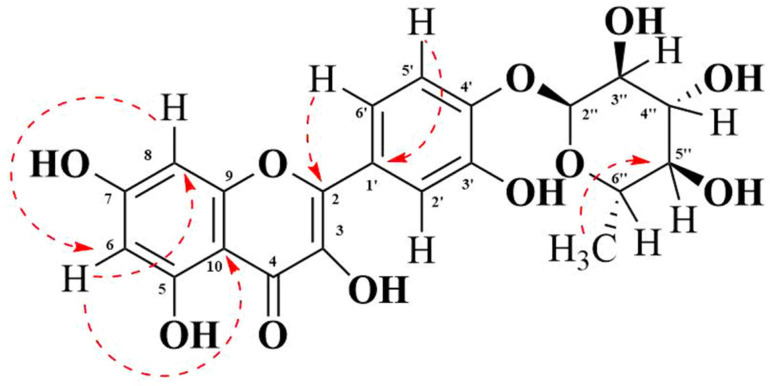
Correlation between the hydrogen atom and carbon. The red dashed arrow indicates the HMBC cross-peak.

Heteronuclear multiple bond correlation spectroscopy (HMBC) shows the correlations between protons and carbons that are separated by multiple bonds (Figure S10). The HMBC spectrum indicated the correlations of the proton at C-6 (δ = 6.38) with the carbon at C-8 (δ = 94.02) and C-10 (δ = 104.5). However, the proton at C-8 (δ = 6.38) correlated only with the carbon at C-6 (δ = 99.1). The proton at C-6′ (δ 7.25) showed correlations with the carbon at C-2 (156.9). It also demonstrated a correlation with the carbon at C-4′ (δ 145.6) and C-2′ (δ 116.04) (not shown in Figure 4). The proton at C-5′ has shown correlation with the carbon at C-1′ (δ 121.50) (Figure 4) as well as with the carbon at C-3′ (δ 145.6) (not shown in Figure 4). Furthermore, the methyl protons at C-7″ (d, 0.807) showed HMBC cross-peaks with the carbon at C-5″ (δ 70.73).

Based on the interactions, as revealed from the COSY and HMBC spectral data and other spectral analyses, the structure of the isolated compound was elucidated as 3,5,7-trihydroxy-2-[3-hydroxy-4-(3,4,5-trihydroxy-6-methyltetrahydro-2H-pyran-2-yloxy)phenyl]-4H-chromen-4-one, which is named folecitin (Figure 2).

### 3.3. Neuroprotective Pharmacology

#### 3.3.1. Folecitin Reduced Oxidative Stress Induced by Ethanol

**Figure 5:**
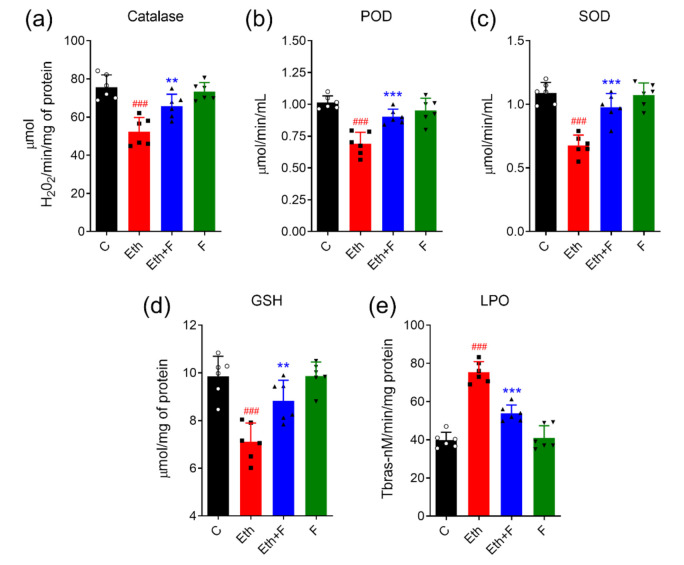
Folecitin abolished ethanol-induced oxidative stress in PND-7 pup brains. Effect of folecitin on the homogenate levels of catalase (**a**), peroxidase (POD) (**b**), superoxide dismutase (**c**), glutathione (GSH) (**d**), and lipid peroxidase (LPO) (**e**) in PND-7 pup brains for the experimental groups, including control (C), ethanol (Eth), Eth + folecitin (F), and folecitin alone. Each bar represents mean levels ± SD (*n* = 6 pups per group). ^###^ *p* < 0.001, as compared to control group; ** *p* < 0.01, *** *p* < 0.001, as compared to the ethanol alone group; one-way ANOVA followed by Tukey’s post hoc test.

The brain homogenates of all rat pups were subjected to different antioxidant assays, such as POD, SOD, CAT, GSH, and TBARS assays. The results revealed that ethanol significantly upgraded oxidative stress by inhibiting SOD (*p* < 0.001), POD (*p* < 0.001), CAT (*p* < 0.01), and GSH (*p* < 0.01) and inducing lipid peroxidase activity (<0.001) in PND-7 rat brains. Interestingly, the co-administration of folecitin significantly restored the activities of antioxidant enzymes SOD (*p* < 0.001), POD (<0.001), CAT (*p* < 0.01), and GSH (*p* < 0.01) while diminishing the activity of the lipid peroxidase (*p* < 0.01) (Figure 5).

#### 3.3.2. Folecitin Inhibited Neuroinflammatory Markers

**Figure 6:**
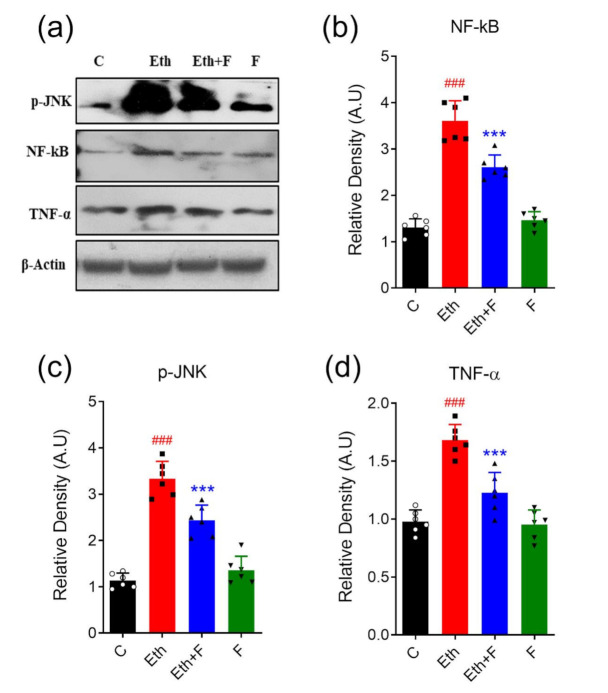
Folecitin inhibited neuroinflammatory markers in PND-7 pup brains. (**a**) Western blots of markers of neuroinflammation, including p-JNK, NF-kB, and TNF-α in the brain homogenates of PND-7 pups for the experimental groups, including control (C), ethanol (Eth), Eth + folecitin (F), and folecitin alone. Histograms of NF-kB (**b**), p-JNK (**c**), and TNF-α (**d**). The bands were quantified using Image J software, and density histograms (expressed in arbitrary units; AU) relative to control were prepared using GraphPad Prism software. Each bar represents mean ± SD for the indicated markers (*n* = 6 pups per group). ^###^ *p* < 0.001, as compared to control group; *** *p* < 0.001, as compared to the ethanol-alone group; one-way ANOVA followed by Tukey’s post hoc test.

Ethanol is linked with a significant proliferation in the protein expression of markers of neuroinflammation, i.e., p-JNK (*p* < 0.001), TNF-α (*p* < 0.05), and NF-kB (*p* < 0.001) in all experimental pups. However, the treatment of pups with folecitin not only significantly inhibited the expression of NF-kB (*p* < 0.001) and p-JNK (*p* < 0.001) but also significantly decreased TNF-α expression (*p* < 0.05) in the brain homogenates of the pups, as shown in Figure 6.

#### 3.3.3. Folecitin Deactivated the NLRP-3 Inflammasome Complex

**Figure 7:**
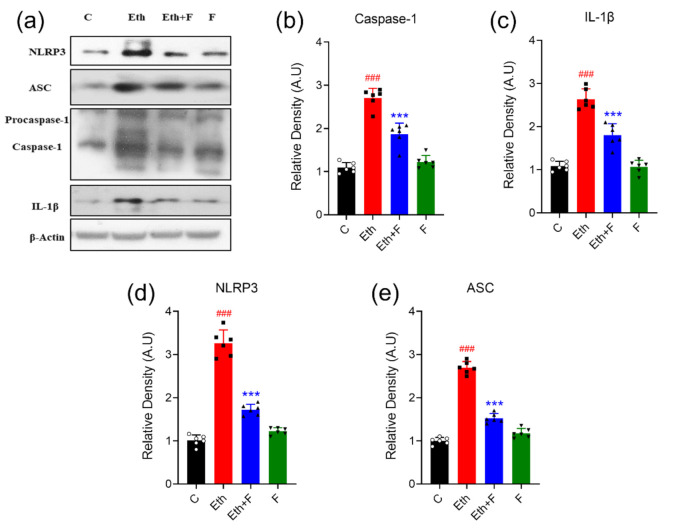
Folecitin deactivated NLRP3 inflammasome complex in PND-7 pup brains. (**a**) Western blots of the NLRP3 inflammasome complex, including NLRP-3, ASC, caspase-1, IL-1β, β-actin in the homogenates of PND-7. pup brains for the experimental groups, including control (C), ethanol (Eth), Eth + folecitin (F), and folecitin alone. Histograms of caspase-1 (**b**) and IL-1β (**c**) and NLRP3 (**d**) and ASC (**e**). The bands were quantified using Image J software, and density histograms (expressed in arbitrary units; AU) relative to control were prepared using GraphPad Prism software. Each bar represents mean ± SD for the indicated proteins (*n* = 6 pups per group). ^###^ *p* < 0.001, as compared to the control group; *** *p* < 0.001, as compared to the ethanol-alone group; one-way ANOVA followed by Tukey’s post hoc test.

In the ethanol-alone-administered groups, a significant increase in the expression of the NLRP-3 inflammasome complex, including NLRP-3 (*p* < 0.001), ASC (*p* < 0.001), caspase-1 (*p* < 0.001), and IL-1β (*p* < 0.001) proteins, was detected in the homogenates of PND-7 rat brains. Co-treatment with folecitin significantly inhibited the exaggerated protein expression of NLRP-3 (*p* < 0.001), ASC (*p* < 0.001), caspase-1 (*p* < 0.001), and IL-1β (*p* < 0.01) in the homogenates of PND-7 rat brains, as shown in Figure 7.

#### 3.3.4. Folecitin Reversed the Expression of the Neuro-Apoptotic Proteins

**Figure 8:**
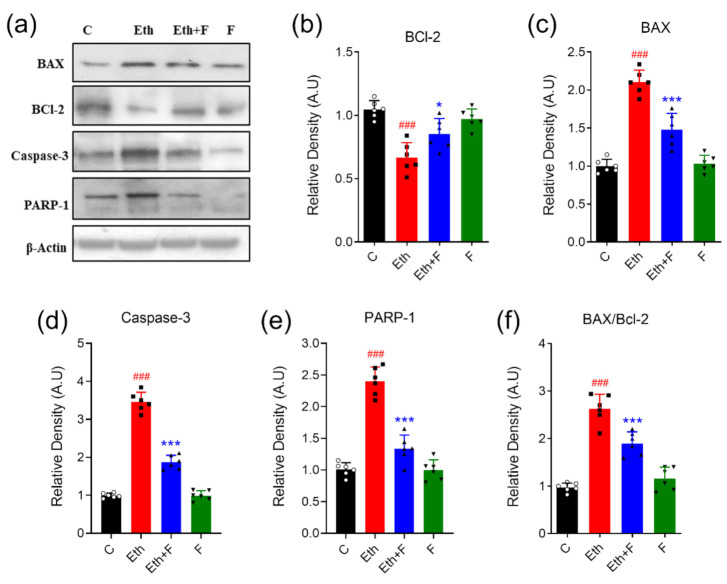
Folecitin reduced ethanol-induced neurodegeneration in PND-7 pup brains. (**a**) Immune-blots of neurodegeneration markers, including BAX, BCL-2, caspase-3, and PARP-1, in the brain homogenates of PND-7 pups for the experimental groups, including control (C), ethanol (Eth), Eth + folecitin (F), and folecitin alone. β-actin was used as the loading control. Histograms of BCL-2 (**b**), BAX (**c**), caspase-3 (**d**) and PARP-1 (**e**), and the BAX/BCL-2 ratio (**f**). The bands were quantified using Image J software, and density histograms (expressed in arbitrary units; AU) relative to control were prepared using GraphPad Prism software. Each bar represents mean ± SD for the neurodegenerative markers (*n* = 6 pups per group). ^###^ *p* < 0.001, as compared to the control group; * *p* < 0.05, *** *p* < 0.001, as compared to the ethanol-alone group; one-way ANOVA followed by Tukey’s post hoc test.

The protein expression of different apoptotic markers, including BAX, BCL-2 and caspase-3, and PARP-1 in the brain homogenates of PND-7 rat pups was determined through the immunoblotting technique. Ethanol administration was associated with a significant protein expression of BAX (*p* < 0.001), caspase (*p* < 0.001), and PARP-1 (*p* < 0.001), while a significant decrease in the expression of BCL-2 (*p* < 0.001) was observed. Moreover, the ratio of BAX to BCL-2 was significantly augmented (*p* < 0.001) in the brain homogenate of pups that were exposed to ethanol alone. Co-treatment with folecitin significantly reduced the increased protein appearance of BAX (*p* < 0.01), caspase-3 (*p* < 0.001), and PARP-1 (*p* < 0.001). On the other hand, folecitin also significantly enhanced the protein expression of BCL-2 (*p* < 0.001). Furthermore, the BAX to BCL-2 ratio was significantly lessened (*p* < 0.01) after co-treatment with folecitin in the brain homogenates of PND-7 rat pups, as shown in Figure 8.

## 4. Discussion

This study was conducted to study the therapeutic potential of flavonoids, i.e., folecitin, isolated from the ethyl acetate fraction of *H. oblongifolium* against ethanol in PND-7 rat pups. This animal model is a well-known and rapid method of ethanol intoxication [41].

**Figure 9:**
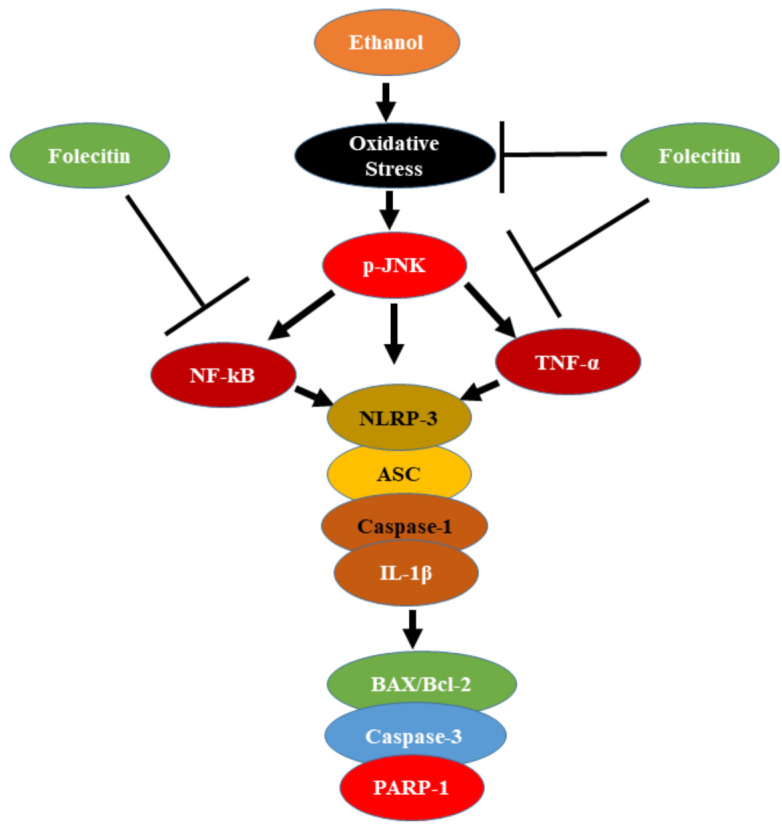
Proposed neuroprotective mechanism of folecitin against ethanol-induced oxidative stress in pup brains. Folecitin in a p-JNK dependent mechanism rescued ethanol-induced neuroinflammation and neurodegeneration.

Neuroprotection against ethanol-induced toxicity via flavonoid therapies has been described in numerous studies [38,42,43,44]. Flavonoid-rich nutrition has been revealed to diminish ethanol-induced impairment to the brain [45]. Similarly, another research finding also confirmed that flavonoids containing supplements prohibited ethanol-encouraged apoptosis in vitro [45,46]. Flavonoids have been highly valued for their incredible antioxidant activity [47,48]. In our current study, a natural antioxidant flavonoid (folecitin) was isolated from the ethyl acetate fraction of *H. oblongifolium* plants, and the structure elucidation of folecitin was done through the techniques of gold-standard routinely used MASS, NMR (COSY, NOESY, and HMBC) spectra [33] and screened to guard the developing brain against the destructive properties of ethanol. Our findings recommend that folecitin overturns ethanol-induced growth in the BAX/BCL-2 ratio and reduces the appearance of stimulated caspase-3 (Figure 9). These results suggest that folecitin can reduce the neurotoxicity caused by ethanol. Interestingly we also found that folecitin significantly reversed the activities of several antioxidant enzymes, such as CAT, POD, GSH, SOD, and LPO. Along with it, folecitin ameliorated the ethanol-induced neuro-apoptotic cascade, neuro-inflammation, and, last but not least, NLRP3 inflammasome complex deactivation in postnatal rat pup brains. To the best of our knowledge, this study is novel because folecitin was used for the first time in such an animal model of neurodegeneration.

Ethanol is a neurotoxin, and animal models suggest that its administration can hurt the brain. This ethanol further triggers unstoppable neuro-inflammation, which results in neuronal death [49]. In this regard, in the CNS, a large quantity of the NLRP3 inflammasome complex occurs [50]. Its main mediators are ROS, neuroinflammatory markers, and endotoxin abundance [51]. As ethanol is responsible for inducing ROS and neuroinflammation, along with other complications in the CNS, it can therefore trigger NLRP3 inflammasome activation in the CNS. Here, in this study, we have observed that with ethanol, after the induction of the oxidative-stress-activated NLRP3 inflammasome complex in PND 7 rat brains. On the other hand, it is worth mentioning that folecitin reduced ethanol-induced oxidative stress and deactivated the NLRP3 inflammasome complex by inhibiting the protein expression of different components of NLRP3, such as ASC and caspase-1, respectively. It is also accompanied by the induction of matured cytokines, i.e., IL-1b, which further causes widespread neuroinflammation and neurodegeneration [52]. This folecitin, through its anti-inflammatory and anti-neurodegenerative capability, significantly abolished the toxic IL-1b and its associated damage to the brains of PND-7 rat pups.

After successful isolation and identification, we have demonstrated in our study that after 4 h of folecitin treatment, the ethanol-induced increased neuroinflammation and ROS production and attenuated neuronal apoptosis in the brain of PND-7 male rat pups is markedly inhibited. This also inhibits the activation of an inflammatory cascade by suppressing activated caspase-3 and TNF-α/NF-κB signaling pathways (Figure 9). The PND-7 developmental stage was chosen because it occurs in the midst of a brain growth spurt period for both rats and mice, and rats at this age have previously shown peak sensitivity to ethanol-induced apoptotic neurodegeneration [23]; male rats were chosen to evade ambiguous sex-dependent differences.

Ethanol administration encouraged the stimulation of the phosphorylated JNK pathway and inflammatory indicators such as TNF-α and NF-κB; co-treatment with folecitin significantly reserved the stimulated NF-κB and TNF-α in the hippocampus of the PND-7 brains (Figure 9). Previous findings also confirmed that ethanol is liable for encouraging inflammation in the CNS [53,54]. For instance, exposure to long-lasting ethanol induces inflammation via the stimulation of NF-κB [55]. Similarly, PND-7 pups exposed to ethanol only once showed substantial neuronal apoptosis within 24 h [56,57]. Compared with the fully-grown brain of adults, the emerging brain is additionally susceptible to neurotoxic damage as it lacks suitable antioxidant enzymatic action [58]. Moreover, the hippocampal and cerebellar areas of the brain are very placid to oxidative stress because of the low levels of vitamin E. Vitamin E has been shown to reverse the stimulation of NF-κB encouraged by ethanol in the hippocampus of PND-7 pup brains [59,60,61].

## 5. Conclusions

In summary, folecitin, being a flavonoid, completely abolished ethanol intoxication in the immature rat brain. This beneficial effect of folecitin (30 mg/kg body weight) is attributed to its antioxidative, anti-neuroinflammatory, anti-apoptotic, and anti-neurodegenerative effects by inhibiting caspase-3, BAX/BCL-2, and PARP-1. Most importantly, the folecitin treatment deactivated the NLRP3 inflammasome complex formation, which depicts its anti-neuro immunological potential. These findings suggest that folecitin could be a novel, safe, and readily available therapeutic agent in treating NDs. More in-depth research and mechanistic approaches are warranted to precisely know the drug-like capabilities of folecitin in both animal models and cell culture.

## Figures and Tables

**Table 1:** List of primary and secondary antibodies with catalog numbers.

S.#	Antibodies Name	Catalogue #
1	Anti-PARP-1	sc-8007
2	Anti-NLRP3	ab270449
3	Anti-TNFα	sc-52746
4	Anti-NF-kB	sc-8008
5	Anti-Caspase-1	sc-56036
6	Anti-Caspase-3	sc-7272
7	Anti-IL-1β	sc-32294
8	Anti-BAX	sc-7480
9	Anti- BCL-2	sc-7382
10	Anti-ASC	sc-514414
11	Anti-p-JNK	sc-6254
12	Anti-beta actin	sc-47778
13	Goat anti-mouse (IgG-HRPs) secondary antibodies	sc-2031

**Table 2:** Yield and Physical Characteristics of Folecitin.

Traits	Results/Description
Physical state	Yellowish-brown crystalline solid powders
UV activity	UV active over TLC
R*f* value	0.4 [methanol (1): ethyl acetate (9)]
Molecular formula	C_21_H_20_O_11_
Crude extract	400 g (ethyl acetate fraction)
Isolated quantity	75 mg
Yield (kg/crude extract)	175
Melting point (°C)	187–189
Solubility	Sparingly soluble in methanol at room temperature

## Data Availability

The authors declare that the data supporting the findings of this study are available within the article or supplementary files.

## Supplementary Materials

The following are available online at https://www.mdpi.com/article/10.3390/life11080825/s1, Table S1: ^1^H and ^13^C-NMR data of compound **1**; Table S2: Figure 6 densitometry readings/intensity ratio; Table S3: Figure 7 densitometry readings/intensity ratio; Table S4: Figure 8 densitometry readings/intensity ratio; Figure S1: Mass spectrum of compound **1**; Figure S2: IR spectrum of compound **1**; Figure S3: ^1^H NMR spectrum of compound **1**; Figure S4: ^1^H NMR spectrum of compound **1**; Figure S5: ^13^C NMR spectrum of compound **1**; Figure S6: ^13^C DEPT 135 NMR spectrum of compound **1**; Figure S7: ^13^C DEPT 90 NMR spectrum of compound **1**; Figure S8: COSY spectrum of compound **1**; Figure S9: HSQC spectrum of compound **1**; Figure S10: HMBC spectrum of compound **1**; Figure S11: Figure S11. Uncropped Western blots of Figure 6 (Folecitin inhibited neuroinflammatory markers in PND-7 pup brains); Figure S12: Uncropped Western blots of Figure 7 (Folecitin deactivated NLRP3 inflammasome complex in PND-7 pup brains); Figure S13: Uncropped Western blots of Figure 8: Folecitin reduced ethanol-induced neurodegeneration in PND-7 pup brains.
