# Supplementary material for: Isolation, Characterization and Neuroprotective Activity of Folecitin: An In Vivo Study
Source: Life (Basel). 2021 Aug 12;11(8):825. doi: 10.3390/life11080825 (PMC8400650; doi:10.3390/life11080825)
Supplement: Supplementary file 1 [file life-11-00825-s001.zip › life-1326366-supplementary.pdf]

life

MDPI

Table S1. ′H and ″C-NMR data of compound 1.

POSITION	′H	″C
1		
2		156.9
3		134.60
4		178.14
5		161.7
6	6.19 (d, = 2.0 Hz)	99.1
7		145.6
8	6.38 (d, = 2.0 Hz)	94.02
9		
10		104.5
1′		121.50
2′	7.30 (d, = 2.0 Hz)	116.04
,	-	145.6
4′	-	148.83
,	6.90 (d, *J* — 8.0 Hz)	115.85
6′	7.25 (dd, *J* — 8.0, 2.0 Hz)	121.11
1″		
2″	5.24 (d, = 1.5 Hz)	102.22
3″	4.59 (dd, *J* — 3.5, 1.5 Hz)	70.44
4″	3.23 (m)	71.56
5″	3.50 (dd, *J* — 9.5, 3.5 Hz)	70.73
	3.49 (m)	70.98
,,	0.807 (d, = 6.0 Hz)	17.90

Table S2. Figure 6 Densitometry readings/intensity ratio.

pJNK				NFKB				TNF-α			
**C**	**Eth**	**Eth+F**	**F**	**C**	**Eth**	**Eth+F**	**F**	**C**	**Eth**	**Eth+F**	**F**
**1.05**	**3.7**	**2.7**	**2**	**1.05**	**4**	**2.6**	**1.6**	**1.05**	**1.6**	**1.1**	**1.2**
**0.95**	**3.6**	**2.5**	**2.1**	**0.95**	**3.9**	**2.5**	**1.5**	**0.95**	**1.7**	**1.2**	**1.25**
**1**	**3.8**	**2.4**	**1.9**	**1**	**4.1**	**2.7**	**1.7**	**1**	**1.5**	**1.15**	**1.1**

Table S3. Figure 7 Densitometry readings/intensity ratio.

	C			Eth			Eth+F			F		
**NLRP3**	**1**	**1.05**	**0.95**	**3.5**	**3.4**	**3.3**	**1.9**	**1.8**	**1.7**	**1.1**	**1.2**	**1.3**
**ASC**	**1**	**1.05**	**0.95**	**2.7**	**2.8**	**2.6**	**1.6**	**1.5**	**1.4**	**1.05**	**1.25**	**1.35**
**Caspase-1**	**1**	**1.05**	**0.95**	**3**	**2.9**	**2.8**	**1.1**	**1.2**	**1.3**	**1.6**	**1.5**	**1.7**
**IL-1β**	**1**	**1.05**	**0.95**	**2.5**	**2.4**	**2.6**	**1.3**	**1.2**	**1.4**	**1.1**	**1.15**	**1.25**

Table S4. Figure 8 Densitometry readings/intensity ratio.

	**BCL-2**							**BAX**			
**C**	**Eth**	**Eth+F**	**F**				**C**	**Eth**	**Eth+F**	**F**	
**1.05**	**0.5**	**1**	**0.9**				**1.05**	**2**	**1.4**	**1.2**	
**0.95**	**0.6**	**0.8**	**0.85**				**0.95**	**2.1**	**1.2**	**1.25**	
**1**	**0.4**	**0.7**	**0.75**				**1**	**2.2**	**1.3**	**1.1**	
	**C**			**Eth**			**Eth+F**			**F**	
**1**	**1.05**	**0.95**	**3.5**	**3.4**	**3.3**	**1.9**	**1.8**	**1.7**	**1**	**0.9**	**0.8**
**1**	**1.05**	**0.95**	**2.1**	**2.2**	**2**	**0.9**	**1**	**0.85**	**0.6**	**0.7**	**0.8**
	**BAX/BCL-2**										
**C**	**Eth**	**Eth+F**	**F**								
**1.05**	**2.5**	**2**	**1.7**								
**0.95**	**2.6**	**2.1**	**1.5**								
**1**	**2.7**	**2.2**	**1.4**								

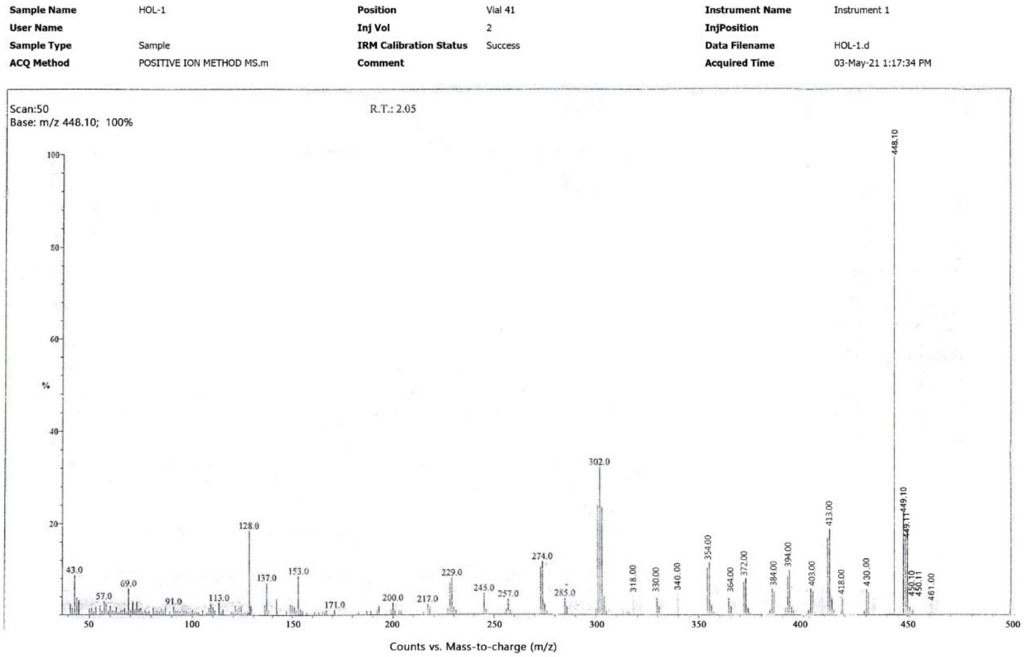

Figure S1. Mass spectrum of compound 1.

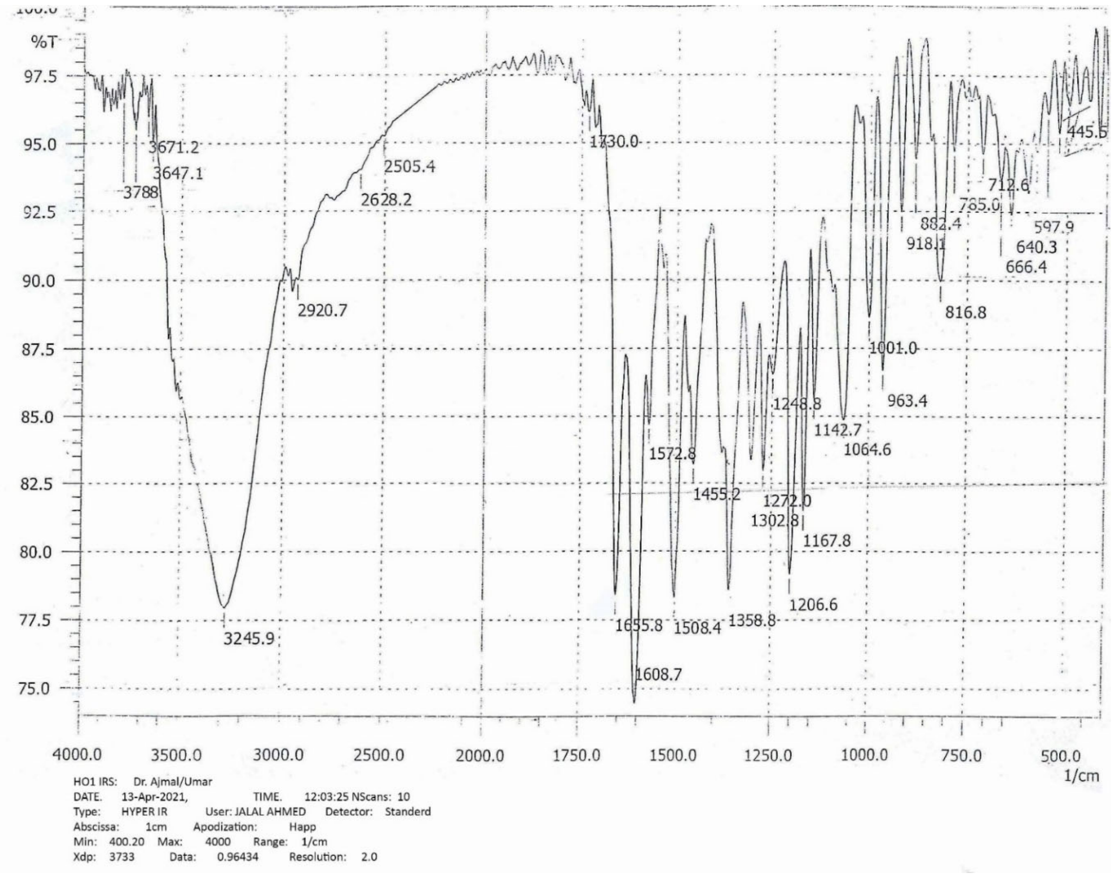

Figure S2. IR spectrum of compound 1.

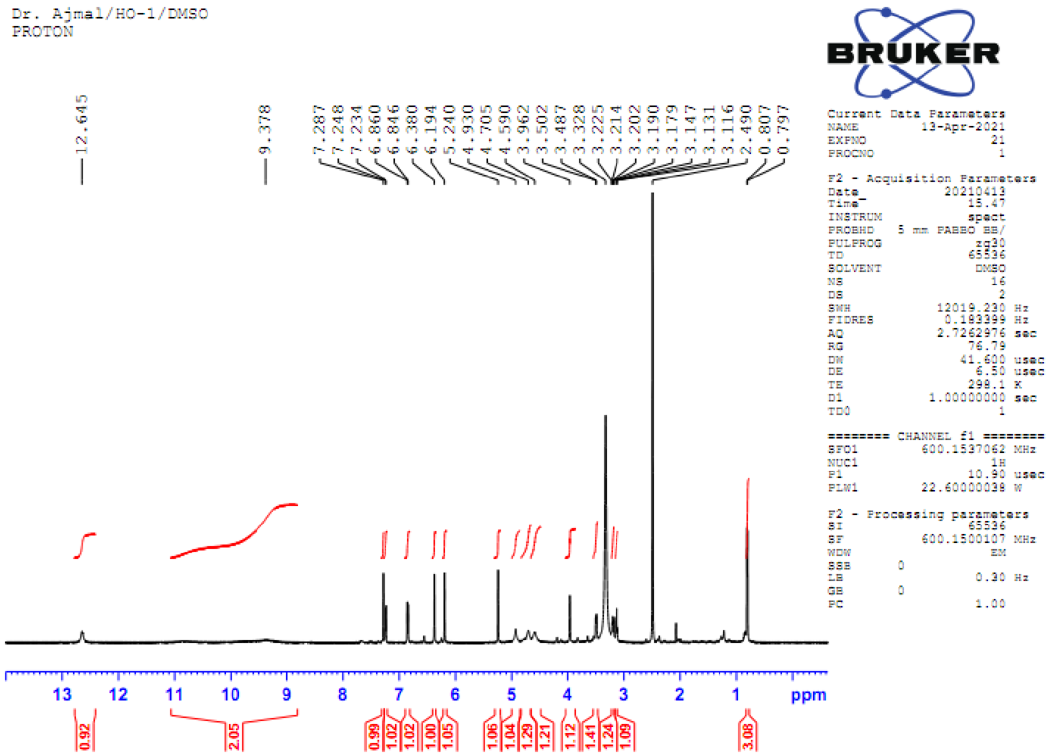

Figure S3. ^{1}H NMR spectrum of compound **1.**

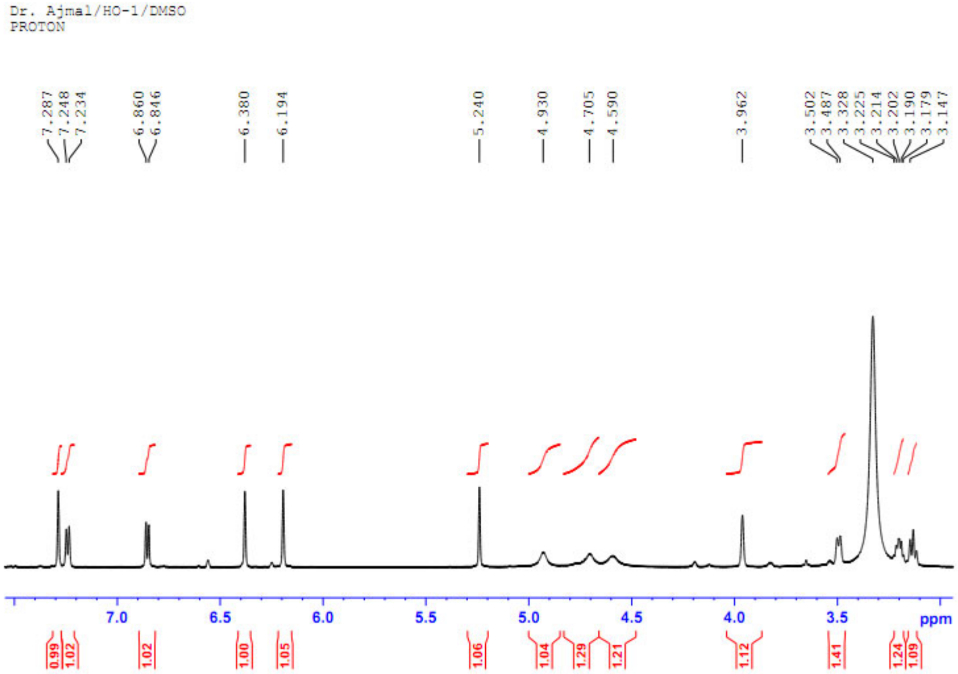

Figure S4. ^{1}H NMR spectrum of compound **1.**

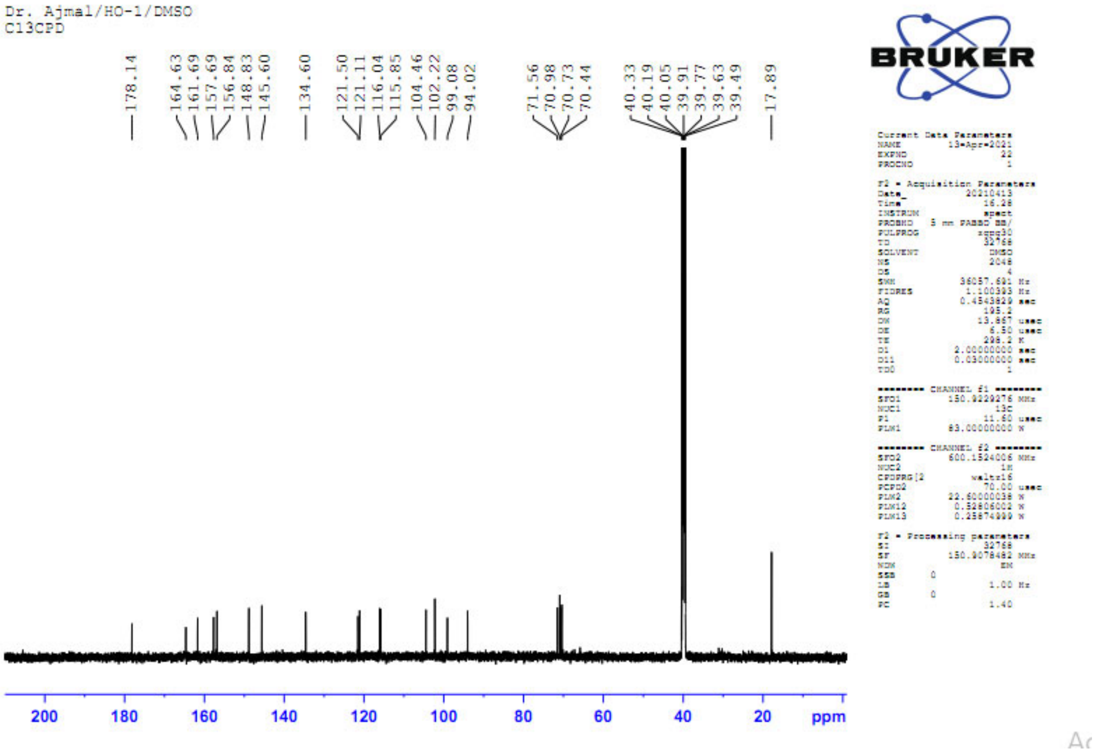

Figure S5. ^{13}C NMR spectrum of compound**1.**

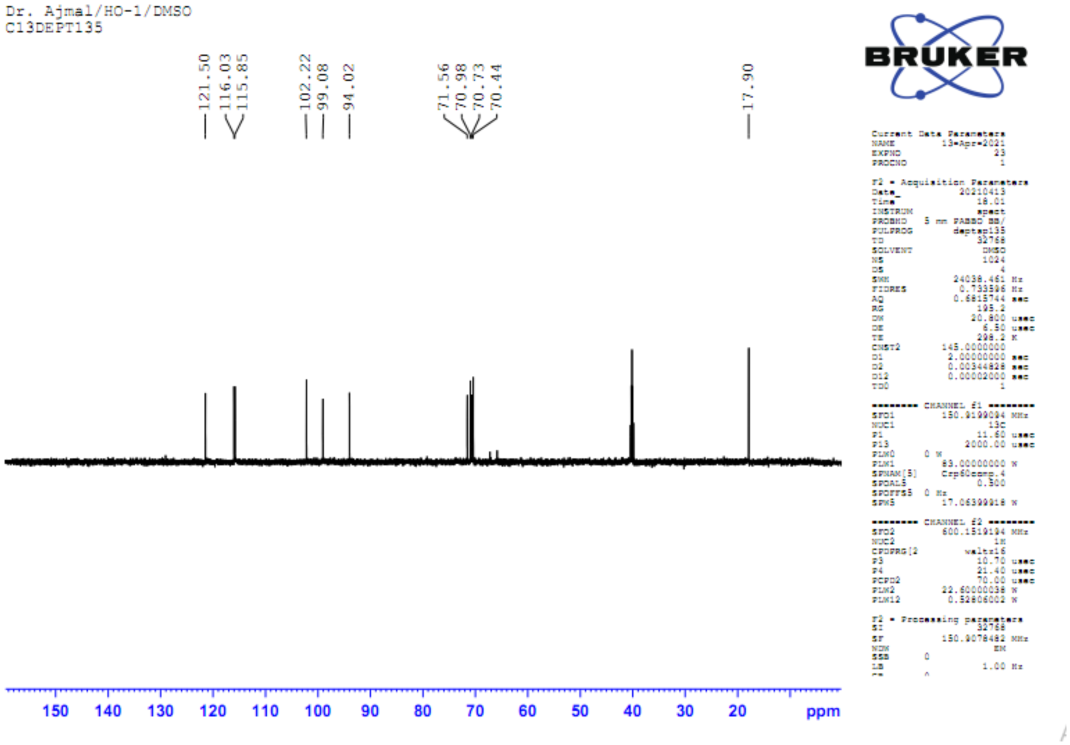

Figure S6. 13C DEPT 135 NMR spectrum of compound 1.

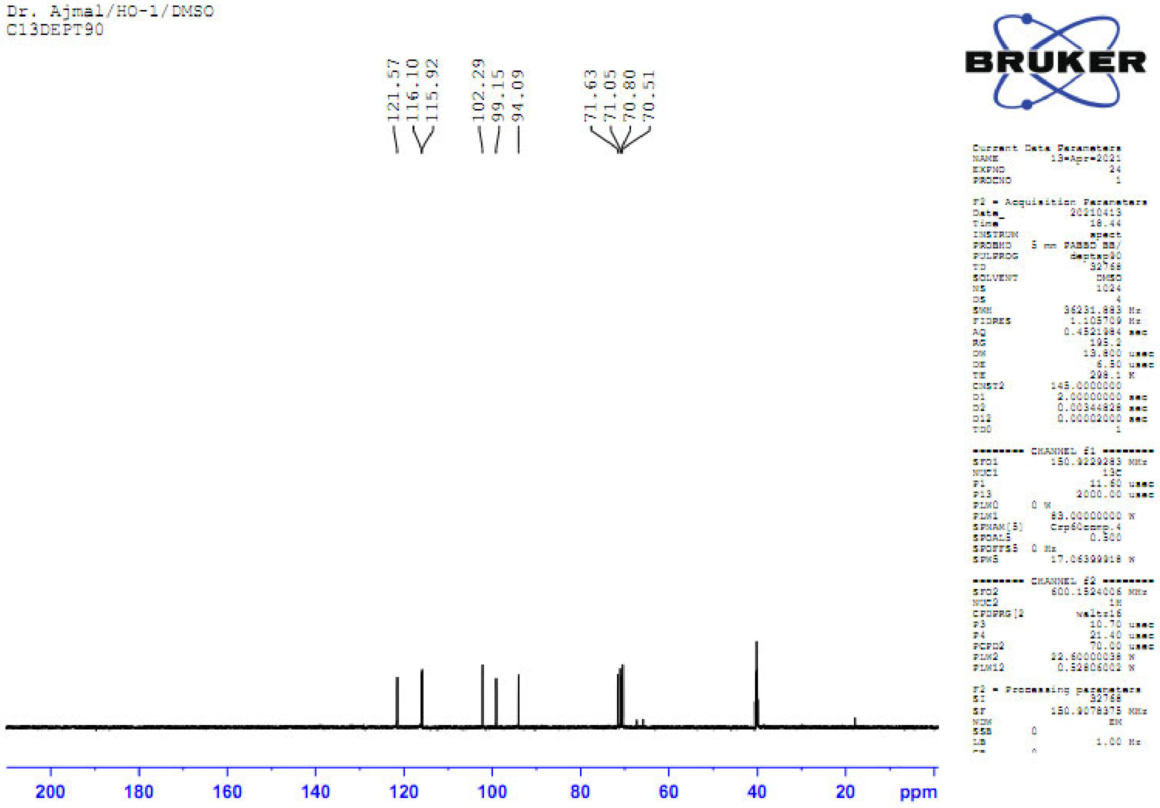

Figure S7. ^{13}C DEPT 90 NMR spectrum of compound**1.**

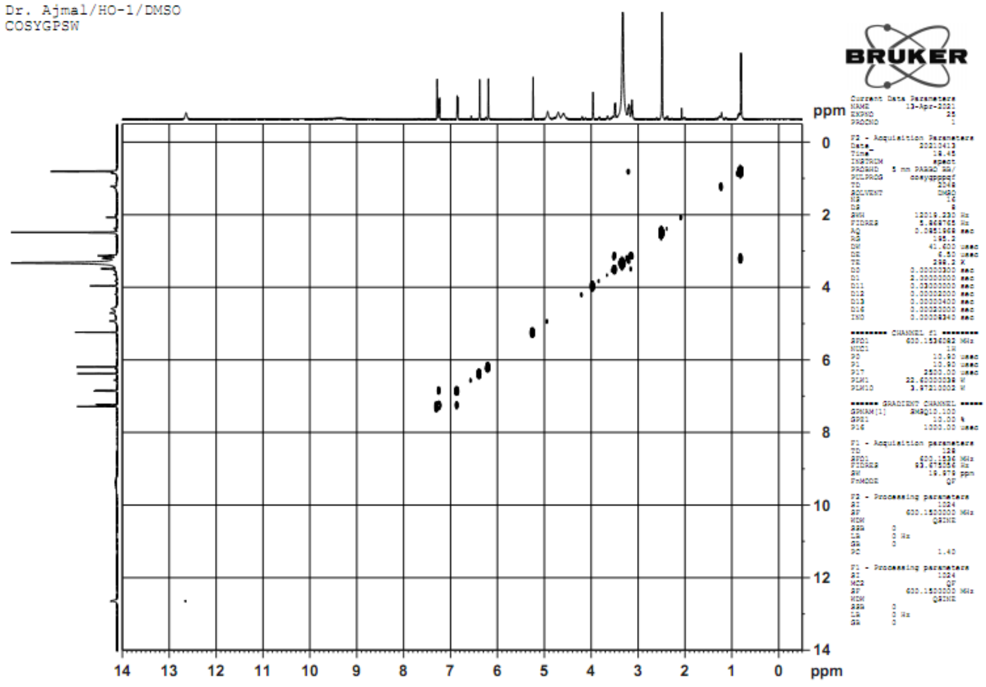

Figure S8. COSY spectrum of compound **1.**

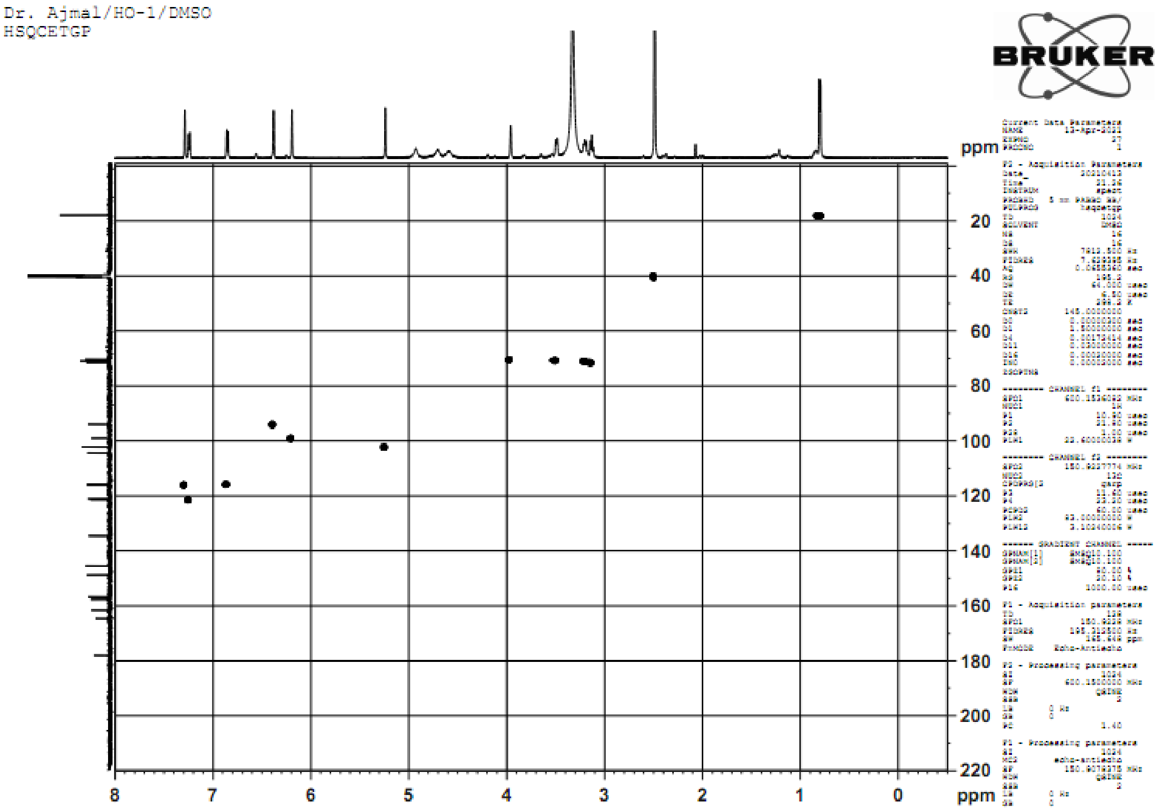

Figure S9. HSQC spectrum of compound **1.**

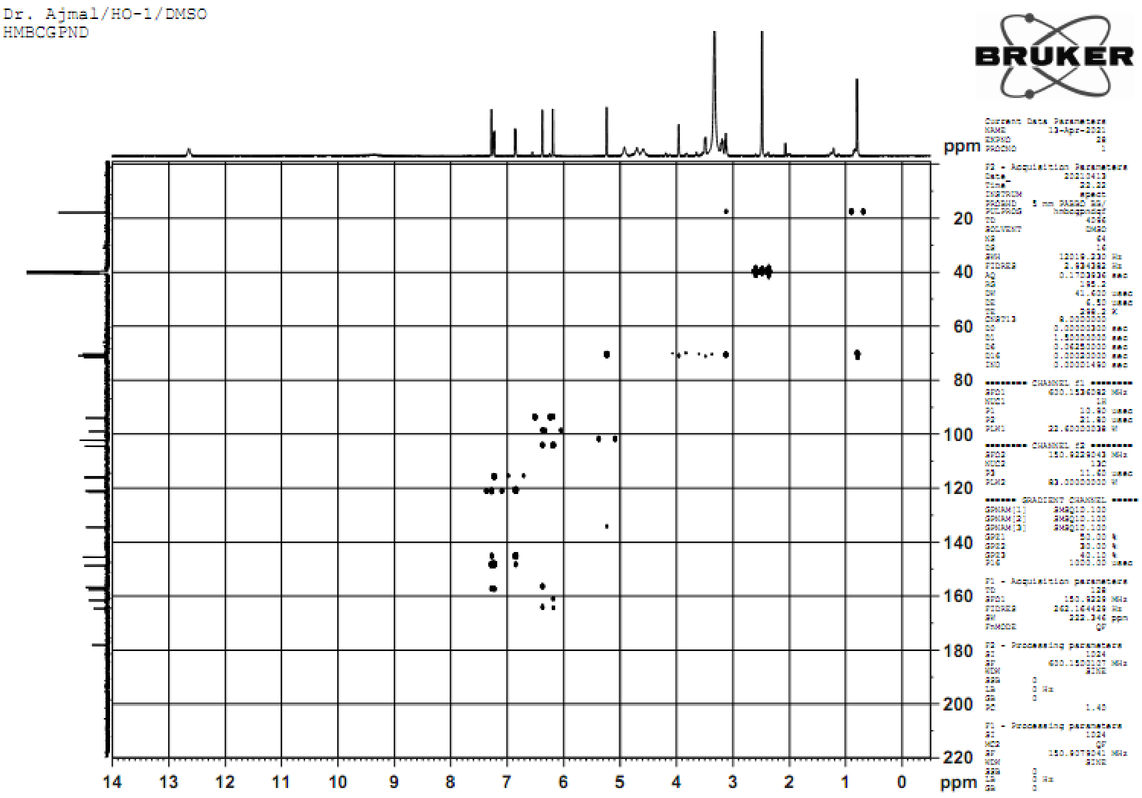

Figure S10. HMBC spectrum of compound **1.**

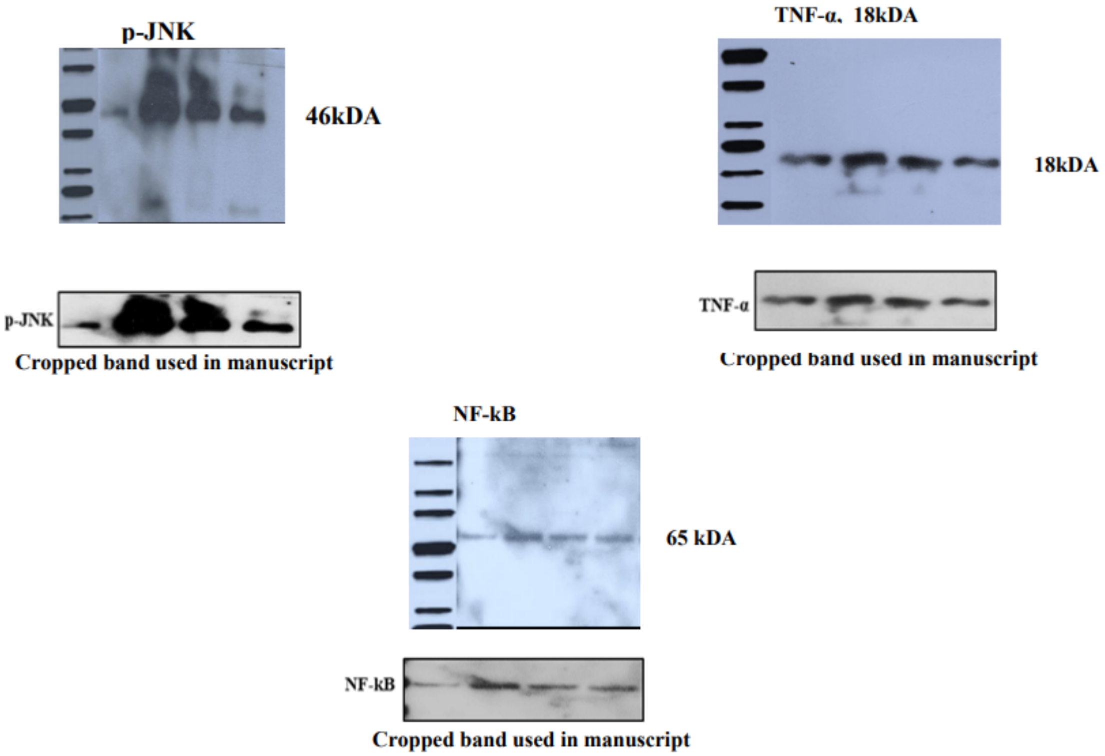

Figure S11. Folecitin inhibited neuroinflammatory markers in PND-7 pup brains.

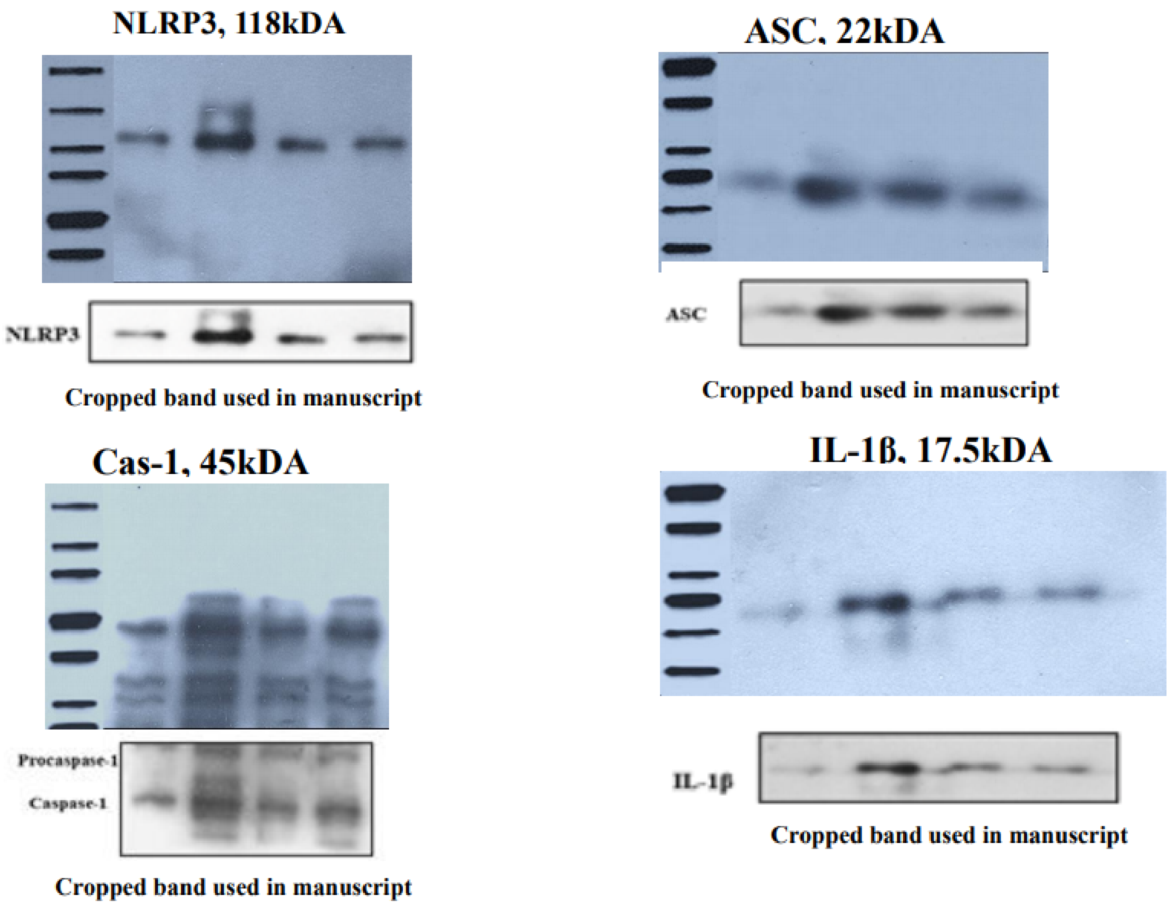

Figure S12. Figure 7. Folecitin deactivated NLRP3 inflammasome complex in PND-7 pup brains.

BAX, 22kDA

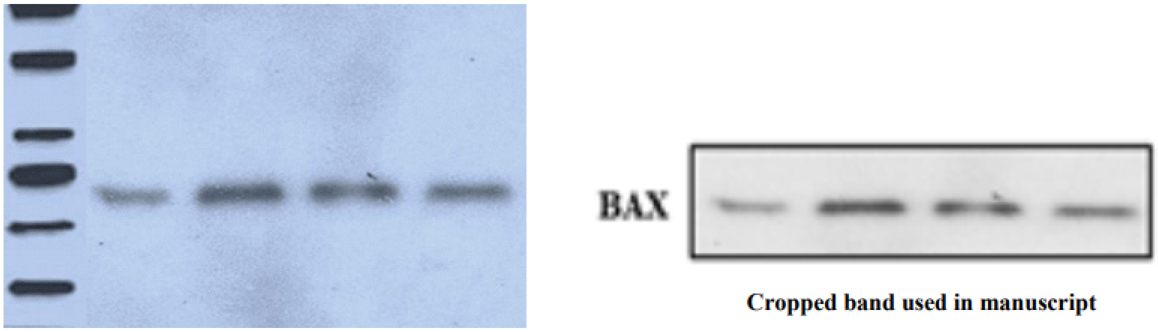

BCl-2, 26kDA

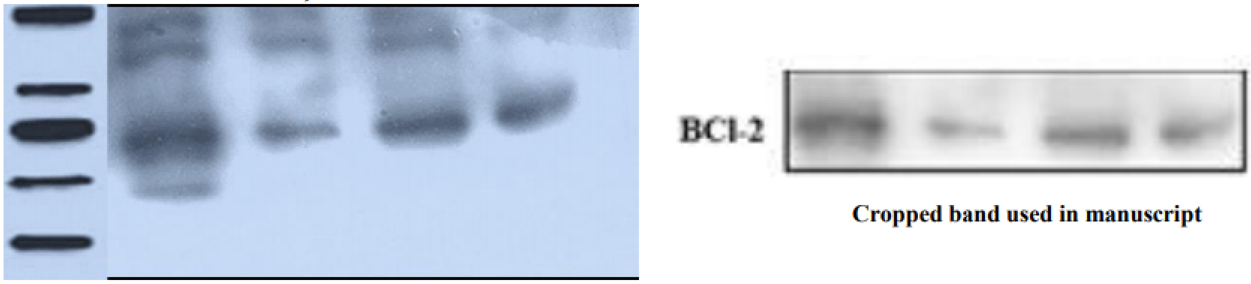

PARP-1, 116/86kDA

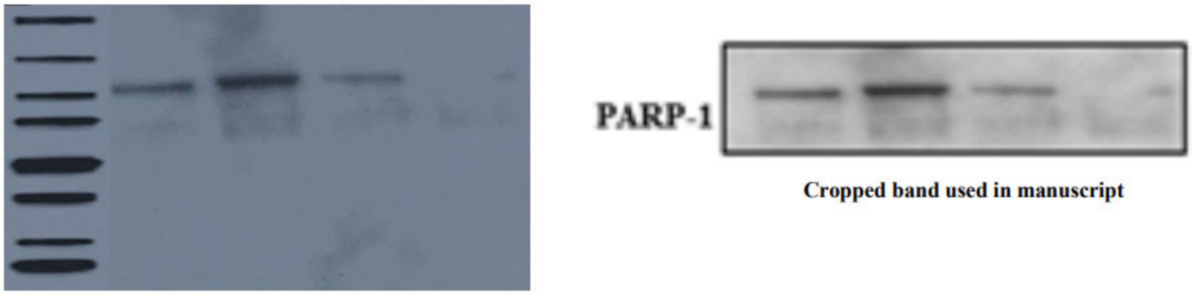

Cas-3, 35kDA

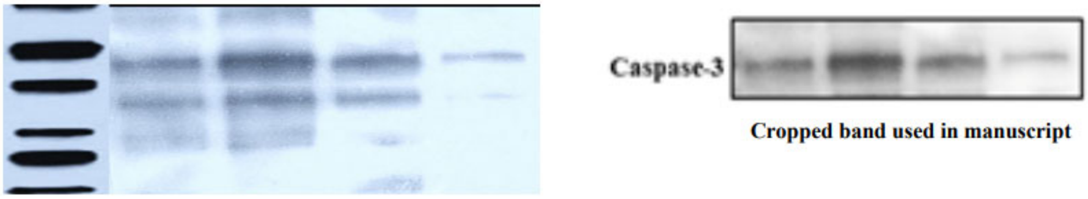

Figure S13. Figure 8. Folecitin reduced ethanol-induced neurodegeneration in PND-7 pup brains.
